# The risk of treatment-emergent affective switch during non-invasive neuromodulation techniques in bipolar depression - A literature review

**DOI:** 10.1192/j.eurpsy.2026.11183

**Published:** 2026-07-31

**Authors:** O. Vasiliu, A. M. Ciobanu, B. M. Petrescu, A. G. Mangalagiu

**Affiliations:** 1 Clinical Neurosciences Department, “Carol Davila” University of Medicine and Pharmacy; 2 Psychiatry Department, “Dr. Carol Davila” University Emergency Central Military Hospital; 3 ”Prof. Al. Obregia” Psychiatry Clinical Hospital, Bucharest, Romania

## Abstract

**Introduction:**

The risk of the treatment-emergent affective switch (TEAS) in bipolar depression (BD) during antidepressant therapy (pharmacotherapy, light therapy, neuromodulation, etc.) is a topic of controversy in the literature. Transcranial magnetic stimulation (TMS) and transcranial direct current stimulation (tDCS) have been explored as an add-on to pharmacological treatment in cases of BD with partial response or non-responsiveness. Electroconvulsive therapy (ECT) is another non-invasive neuromodulation technique that has been used in clinical practice for more than eight decades, and is supported by a high level of evidence in the treatment of both manic and depressive episodes of bipolar disorder.

**Objectives:**

The objective of this research was to explore the currently available level of evidence for the existence of TEAS in patients diagnosed with BD who are undergoing non-invasive neuromodulation treatment.

**Methods:**

A literature review was conducted in three electronic databases (PubMed, Cochrane Library, and Clarivate/Web of Science) using the keywords „bipolar depression,” „treatment-emergent affective switch,” „tDCS,” „TMS,” “ECT,” and „neurostimulation” for papers published in English between January 2000 and February 2025.

**Results:**

Twenty-one primary and secondary reports of adult patients with BD were identified and further explored. Episodes of TEAS were reported during tDCS and rTMS, although the reported incidence was low or very low when compared to placebo. Protocols of repetitive TMS applied on the right/left/bilateral dorsolateral prefrontal cortex were evaluated, while the protocols for tDCS were less standardized. The duration and frequency of the sessions were very heterogeneous, making the interpretation of results more challenging. ECT was associated with an extremely variable rate of TEAS, varying from 0 to 24.8% in clinical studies, depending on the methodology used. There is a need for sham-controlled trials with more homogeneous populations, but based on the existing data, TEAS does not appear to be a relevant source of treatment discontinuation in patients with bipolar disorders.

**Conclusions:**

Based on the current evidence (low to moderate quality of data), non-invasive neuromodulation interventions are relatively safe from the TEAS in patients with BD when used as an add-on to pharmacological therapy. However, since TEAS has been reported in patients with ECT, and is extremely rare in patients with tDCS and rTMS, patients undergoing these types of therapy should be monitored for the potential occurrence of mood switch.

**Disclosure of Interest:**

None Declared

